# Quantitative high-throughput phenotypic screening of pediatric cancer cell lines identifies multiple opportunities for drug repurposing

**DOI:** 10.18632/oncotarget.23462

**Published:** 2017-12-19

**Authors:** Min Shen, Rosita Asawa, Ya-Qin Zhang, Elizabeth Cunningham, Hongmao Sun, Alexander Tropsha, William P. Janzen, Eugene N. Muratov, Stephen J. Capuzzi, Sherif Farag, Ajit Jadhav, Julie Blatt, Anton Simeonov, Natalia J. Martinez

**Affiliations:** ^1^ National Center for Advancing Translational Sciences, National Institutes of Health, Rockville, MD, USA; ^2^ Division of Pediatric Hematology Oncology, University of North Carolina, Chapel Hill, NC, USA; ^3^ Lineberger Comprehensive Cancer Center, University of North Carolina, Chapel Hill, NC, USA; ^4^ Eshelman School of Pharmacy, University of North Carolina, Chapel Hill, NC, USA; ^5^ Epizyme, Inc., Cambridge, MA, USA

**Keywords:** drug repurposing, quantitative high-throughput screening, pediatric cancer, 3D cultures

## Abstract

Drug repurposing approaches have the potential advantage of facilitating rapid and cost-effective development of new therapies. Particularly, the repurposing of drugs with known safety profiles in children could bypass or streamline toxicity studies. We employed a phenotypic screening paradigm on a panel of well-characterized cell lines derived from pediatric solid tumors against a collection of ∼3,800 compounds spanning approved drugs and investigational agents. Specifically, we employed titration-based screening where compounds were tested at multiple concentrations for their effect on cell viability. Molecular and cellular target enrichment analysis indicated that numerous agents across different therapeutic categories and modes of action had an antiproliferative effect, notably antiparasitic/protozoal drugs with non-classic antineoplastic activity. Focusing on active compounds with dosing and safety information in children according to the Children's Pharmacy Collaborative database, we identified compounds with therapeutic potential through further validation using 3D tumor spheroid models. Moreover, we show that antiparasitic agents induce cell death *via* apoptosis induction. This study demonstrates that our screening platform enables the identification of chemical agents with cytotoxic activity in pediatric cancer cell lines of which many have known safety/toxicity profiles in children. These agents constitute attractive candidates for efficacy studies in pre-clinical models of pediatric solid tumors.

## INTRODUCTION

Drug repurposing or repositioning, the use of drugs for indications other than what they were initially intended, has been offered as a strategy for drug development in oncology programs. This approach has special potential for childhood cancers and other rare or orphan diseases, where the expected return on new drug development has been a disincentive to pharmaceuticals [1, 2]. Historically, oncology agents are approved for pediatric cancers only after efficacy is demonstrated in adult tumors. Hence, repurposing drugs that particularly have known safety profiles in children and adolescents could streamline or directly bypass toxicity studies, making these drugs more appealing to providers and patients [3, 4].

Drug repurposing in childhood cancers may occur in several ways [5]. One serendipitous way is the observation of unintended antineoplastic benefits of drugs that have been prescribed for different indications. This has been the path to repurposing several drugs which now have childhood cancer indications [3, 4, 6, 7]. Another way includes more systematic hypothesis-driven approaches based on mining existing drug and structural databases [8–13]. A third approach to repurposing, which exploits the polypharmacology of drugs to discover new drug indications, is the use of phenotypic screening [14–17]. This approach interrogates drug candidates in pediatric cancer models in an unbiased fashion and has the advantage of providing information on large numbers of drugs in relatively short periods of time. Additionally, identifying approved drugs with previously unrecognized anticancer properties has the potential to reveal new mechanisms and biological processes involved in carcinogenesis or drug resistance.

In this study, we performed phenotypic screening on 19 well-characterized cell lines derived from solid tumors seen in children and adolescents against a collection of 3,886 unique compounds spanning approved drugs and investigational agents [18–21]. Specifically, we employed a titration-based screening paradigm (quantitative high-throughput screening or qHTS), where all compounds were tested at multiple concentrations for their effect on cell viability [22]. Thus, concentration-response curves (CRCs) were derived directly from the primary screen, and both potency and efficacy values were used to identify compounds with robust bioactivity profiles. By combining this approach with available information associated with drug annotations, we found numerous agents across different therapeutic categories and modes of action that had an antiproliferative effect. Among these we found antiparasitic/protozoal and other drugs with non-classic antineoplastic mode of action. We focused validation studies mainly on compounds that are present in our Children's Pharmacy Collaborative (CPC) database, a comprehensive list of drugs with dosing and safety profiles in children (0–18 years old) [4]. Specifically, we implemented a high-throughput multiparametric assay utilizing three-dimensional (3D) cell cultures as *in vitro* models of pediatric cancers to further validate compound activity. Moreover, we show that selected antiparasitic agents induce cytotoxicity by activating apoptosis-mediated cell death.

By combining phenotypic screening data and CPC information, our platform enables the identification of chemical agents with cytotoxic activity in pediatric cancer cell lines as well as known safety/toxicity profiles in children. These agents constitute attractive candidates for efficacy studies in clinical trials of pediatric solid tumors.

## RESULTS

To identify new therapeutic options for pediatric cancer, we screened the NIH Chemical Genomics Center (NCGC) Pharmaceutical Collection (NPC)[21] and Mechanism Interrogation Plate (MIPE) [18–20] small molecule collections of approved and investigational drugs using a 1,536-well format qHTS assay against a panel of pediatric cancer cell lines. The panel consisted of 19 well-characterized cell lines derived from childhood solid tumors, namely Ewing's sarcoma (EWS), central nervous system (CNS) tumors (medulloblastoma, glioblastoma, and atypical teratoid rhabdoid tumor [ATRT]), neuroblastoma (NB), osteosarcoma (OS), and rhabdomyosarcoma (RMS) (Table 1 and Supplementary Table 1). Of note, all cell lines actively proliferate in the time course of the assay (Supplementary Figure 1). The assay measured metabolically active cells after 48 hours of compound treatment as a proxy for cell viability. For each of the 19 cell lines tested, we derived concentration-response curves (CRCs) for 4,728 compounds (comprising 3,886 unique compounds), and we used both potency and efficacy values to identify compounds with robust bioactivity profiles (definition of activity and cutoffs are described in Materials and Methods and assay performance is described in Supplementary Table 2). Briefly, compounds exhibiting high-quality dose-response curves, IC_50_ of ≤ 10 μM, and maximal response ≥ 65% were considered as active. A total of 1,120 compounds were active against one or more cell lines (Supplementary Figure 2A). Unsupervised clustering of pharmacological responses of active compounds indicated that not all cell lines clustered based on their tumor type of origin (Supplementary Figure 2B). This is not surprising given that each tumor type is only represented by a small number of cell lines; also, the artificially grouped “CNS tumor” category contains representative cell lines from three different tumor types. Interestingly, rhabdomyosarcoma lines are among the cell lines with fewer number of hits (most resistant) and EWS lines among the ones with greater number of hits (most sensitive) (Supplementary Figure 2C). A total of 62 compounds, referred herein to as pan-actives, were active across 17 or more cell lines (Supplementary Figure 2D). Target-based analysis of the pharmacological responses indicated an overrepresentation of DNA topoisomerase, histone deacetylase (HDAC), Interleukin-2-inducible T cell kinase (ITK), Janus kinase 2 (Jak2), phosphoinositide 3-kinase (PI3K), and proteasome inhibitors among pan-actives (Supplementary Figure 2E). Interestingly, 36 of these pan-actives had minimal activity (inactive or did not pass potency and/or efficacy cutoffs) against human wild-type fibroblasts (Hh-Wt-fibroblasts) indicating their activity is not the result of non-specific cytotoxic effects.

**Table 1:** Cell lines used in the study

Cell Line		
Tumor Type	Name	Source
EWS	A673	UNC
	TC32	UNC
	SK-N-MC	UNC
CNS: Glioblastoma	SJ-GBM2	PPT
CNS: Medulloblastoma	Daoy	UNC
CNS: ATRT	BT-12	UNC
	BT-37	UNC
NB	LAN-5	UNC
	NB1643	PPT
	NB-EBc1	PPT
	SK-N-SH	UNC
OS	MG 63 (6-TG R)	UNC
	OHS-50	UNC
	Saos-2	UNC
	U-2 OS	UNC
RMS	RD	UNC
	Rh18	UNC
	Rh30	PPT
	Rh41	PPT
Control	Hh-Wt-fibroblasts	CI

EWS: Ewing's sarcoma; CNS: Central Nervous System; ATRT: atypical teratoid rhabdoid tumor; NB: Neuroblastoma; OS: Osteosarcoma; MG 63 (6-TG R): 6-Thioguanine Resistant MG 63 cell line; RMS: Rhabdomyosarcoma. Sources: UNC: University of North Carolina; PPT: Pediatric Preclinical Testing Program; CI: Coriell Institute.

**Figure 1:**
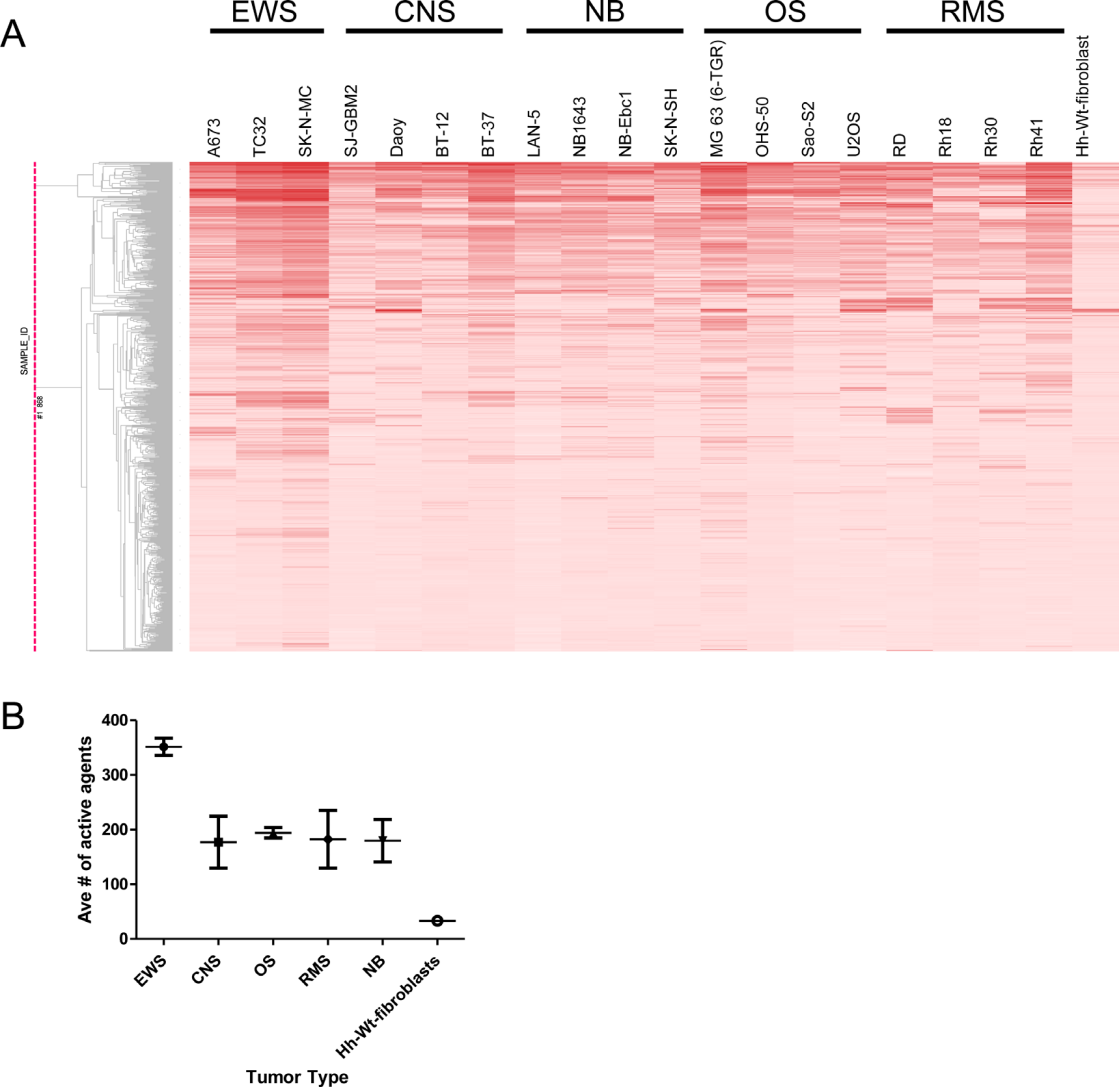

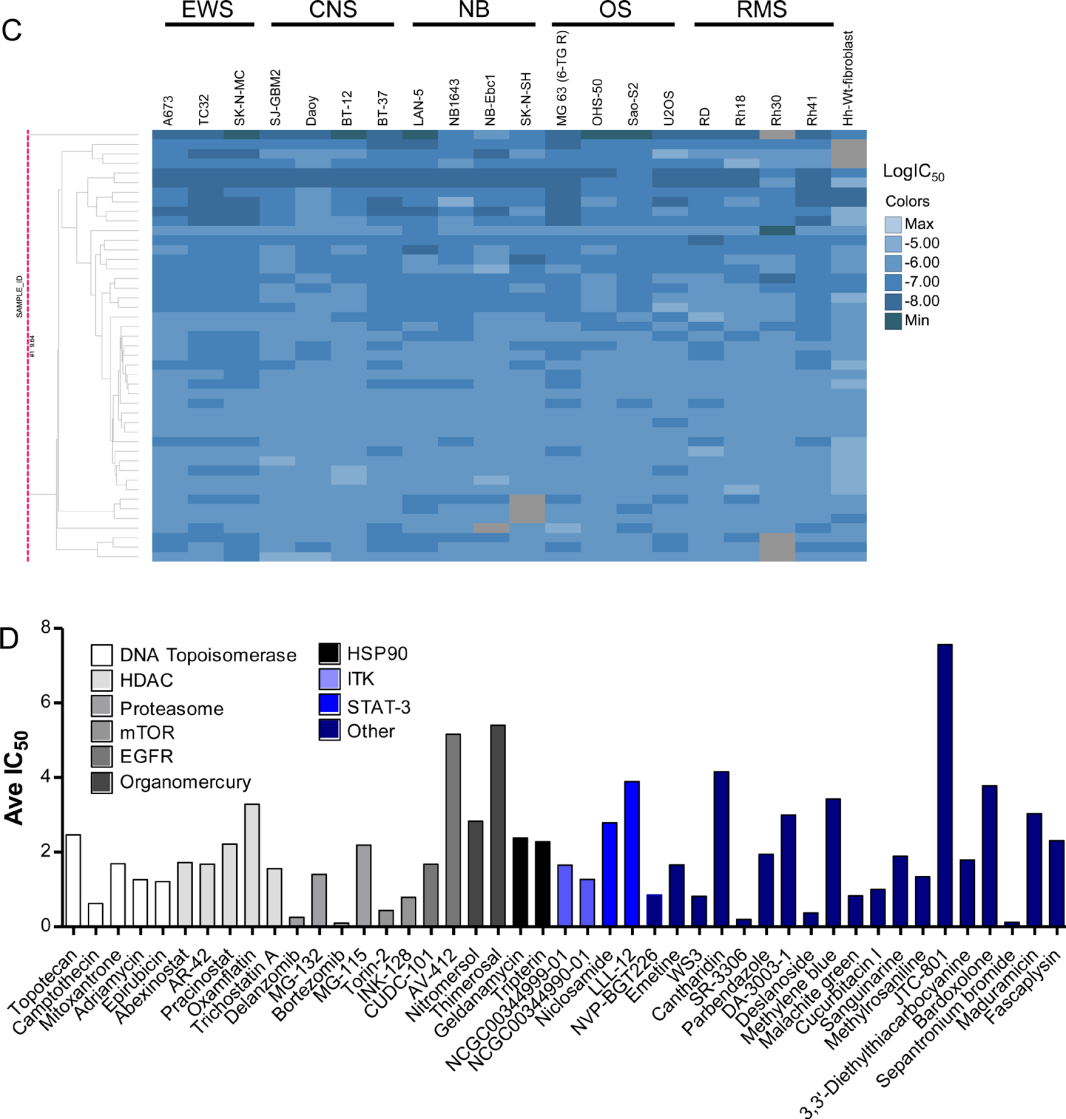
Follow-up studies validate compound activity (**A**) Hierarchical clustering of compound activity rendered by tumor type. Compound activity is represented as AUC (Area Under the Curve) values. AUC values are colored red, with darker color indicating compounds that are more potent and efficacious. (**B**) Distribution of active agents by tumor type. (**C**) Hierarchical clustering of pan-active compounds (active in 17 cell lines or more) based on potency. LogIC_50_ values are colored in blue, with darker color indicating more potent compounds. Inactive compounds that have no IC_50_ values determined are shown in grey. (**D**) Target-based analysis of pan-active compounds in follow-up screen. For each compound, the average IC_50_ across all cell lines in shown.

To confirm activity, 736 compounds were selected for retesting in secondary follow-up screens against the panel of cell lines. Criteria for selection included one or more of the following: availability in our repository; activity against the majority of cell lines in the primary screen (pan-active compounds); selective activity against multiple cell lines within a tumor type (selective compounds); and finally, strong activity (IC_50_ of <100 nM) against at least one cell line (potent compounds). To support our drug repurposing effort the retesting set included compounds covering a diverse range of mechanisms of action and with and without known antineoplastic activity. Using the same activity cutoffs as in the primary screen, of the 736 compounds retested, 502 (68.2%) showed activity against one or more cell lines in any tumor category (Figure 1A, Supplementary Table 3 and Supplementary Table 4). In agreement with the primary screen, cell lines from Ewing's sarcomas were among those with higher number of hits (most sensitive), with approximately 350 active compounds (Figure 1B). In the retest, 45 compounds showed a relatively broad range of *in vitro* activity with cytotoxicity against multiple cell lines (pan-actives, Figure 1C). In concordance with the primary screen, pan-active compounds were enriched for DNA topoisomerase and HDAC inhibitors, and the vast majority either have been or are being investigated for antineoplastic indications (Figure 1D). Dose-response curves of select pan-active compounds are shown in Supplementary Figure 3. Among these pan-actives, 27 compounds exert minimal activity against human fibroblasts cells (Supplementary Figure 3B).

**Figure 2:**
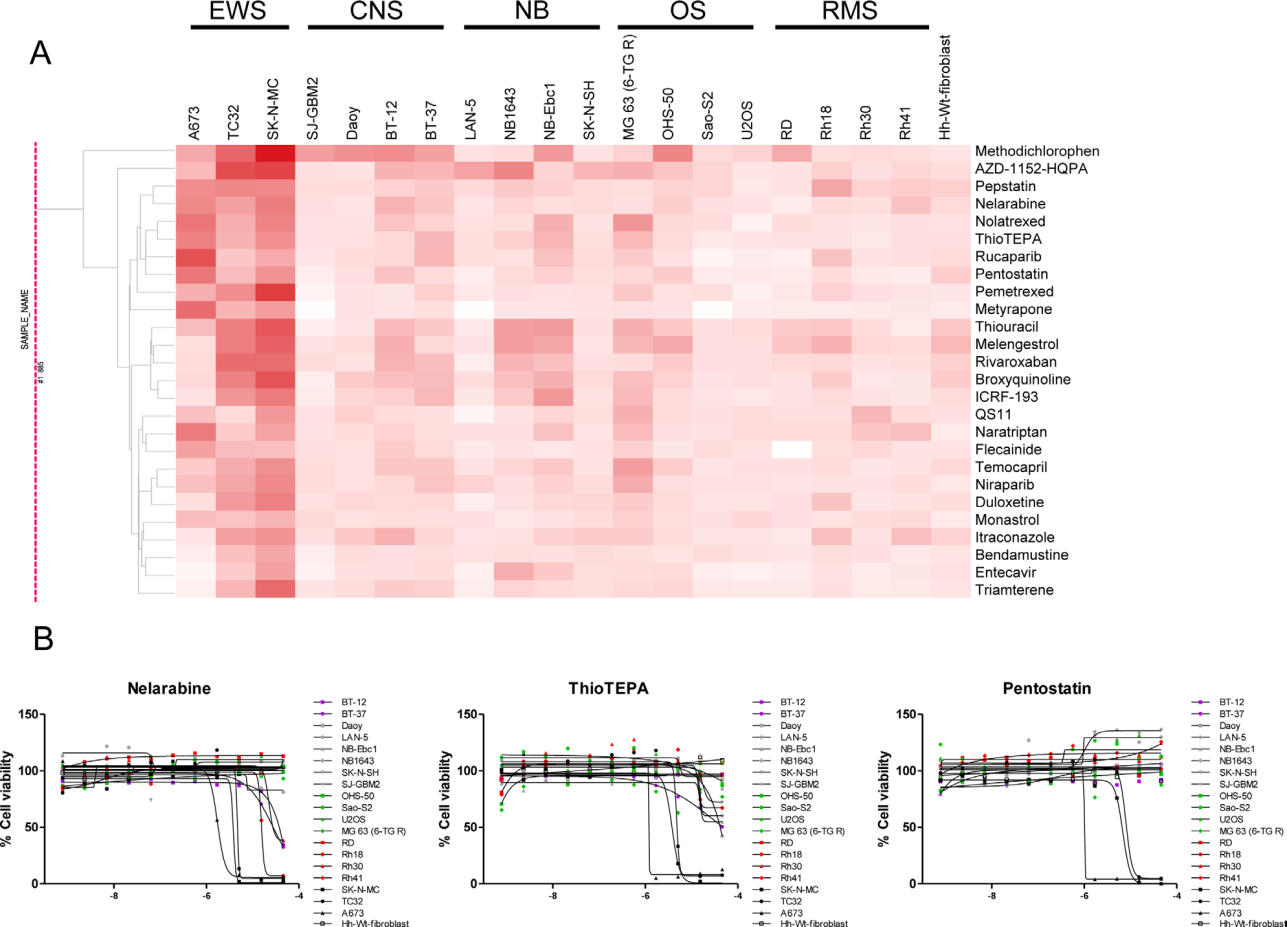
Compounds with specific activity against EWS cancer cell lines (**A**) Clustering of retested compounds rendered by tumor type identifies EWS-specific agents. Hierarchical clustering of compound activity based on AUC (Area Under the Curve) values rendered by tumor type. AUC values are colored red, with darker color indicating compounds that are more potent and efficacious. (**B**) Dose response curves of EWS-specific antineoplastic agents. Exemplified are the purine nucleoside analogs (or antimetabolite) Nelarabine, and Pentostatin and the alkylating agent ThioTEPA.

Activity outcomes of retested compounds rendered by tumor types identified 26 tumor-specific compounds that showed activity against two or more cell lines from the EWS tumor category (Figure 2A and Supplementary Figure 4). While the majority of these compounds have known antineoplastic effects, other have diverse indications such as antivirals, antiprotozoal and antiarrhythmic (Figure 2B and Supplementary Table 5). A recent study by Pessetto *et al.* reported an *in vitro* drug screening of an FDA-approved drug library against a set of EWS cell lines, of which A673 was also screened in our panel [17]. The authors identified 45 drugs with activity against EWS lines and minimal activity against non-tumorigenic control lines. Remarkably, among the 45 hits, 28 compounds were also included in our re-test set and 23 of these were active against EWS lines, including auranofin. However, none of the 23 active agents were specific for EWS lines since they also showed activity against other tumor lines included in our study.

Potency analysis among active compounds in the follow-up screen identified 90 compounds with potencies lower than 100 nM against at least one cell line (Supplementary Table 6). DNA topoisomerase, proteasome, mechanistic target of rapamycin (mTOR) and tubulin polymerization inhibitors were the most represented mechanisms with potent activity (Supplementary Figure 5).

**Figure 3:**
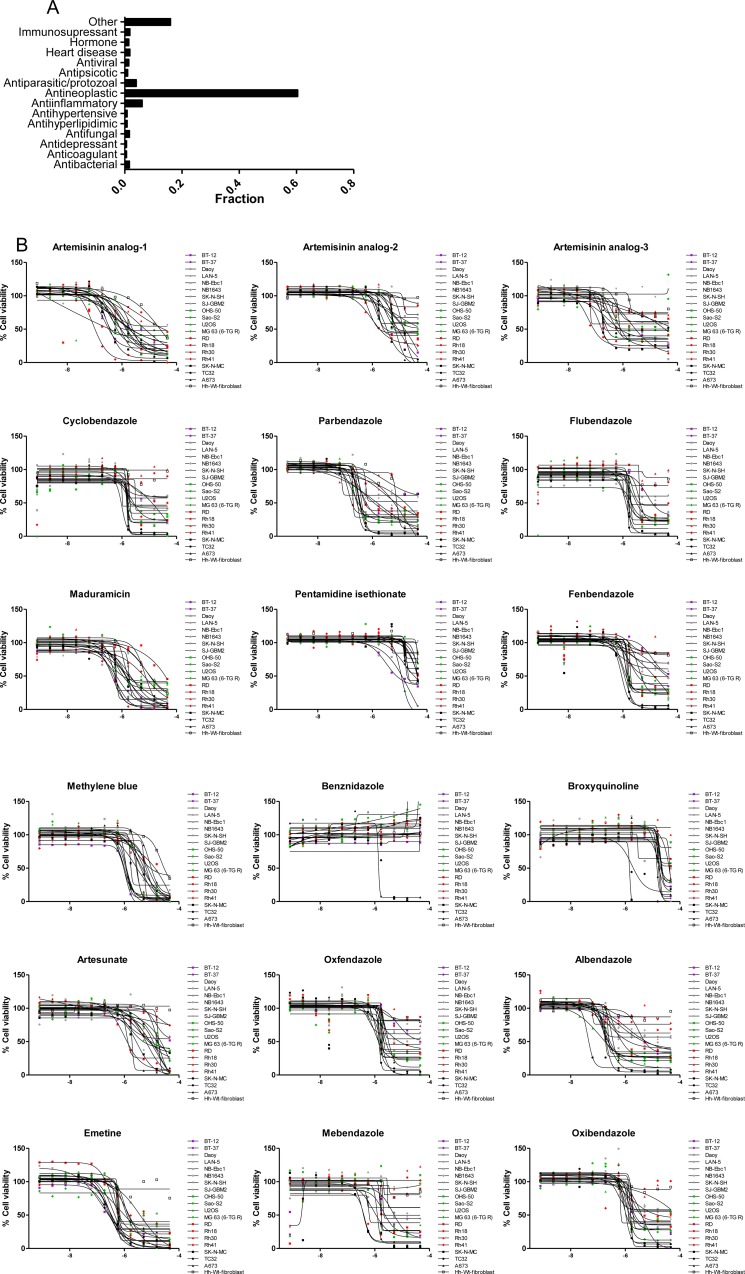
Indication of active compounds identified in the follow-up screen (**A**) Fraction of compounds that are active against one or more cell line. The category labeled as “Other” contains indications represented by 1 compound. (**B**) Dose response of active antiparasitic/protozoal agents. X-axis represents Log[M].

Although the majority (∼58%) of active agents from the follow-up screen have oncology indications or are being studied for antineoplastic effects, ∼42% have a primary indication other than cancer. Among the latter, we found compounds used as antiparasitics and antiprotozoals (Figure 3 and Supplementary Table 7). Antiparasitic/protozoal agents have been hypothesized by others to play anticancer roles [23–26]. In fact, several drugs in clinical use against these diseases, such as mebendazole, are currently in clinical trials for childhood cancers [27]. Similarly, antiinflammatory agents, in particular non-steroidal (NSAIDs) and glucocorticoids, have also been linked to antineoplastic effects [28–30].

**Figure 4:**
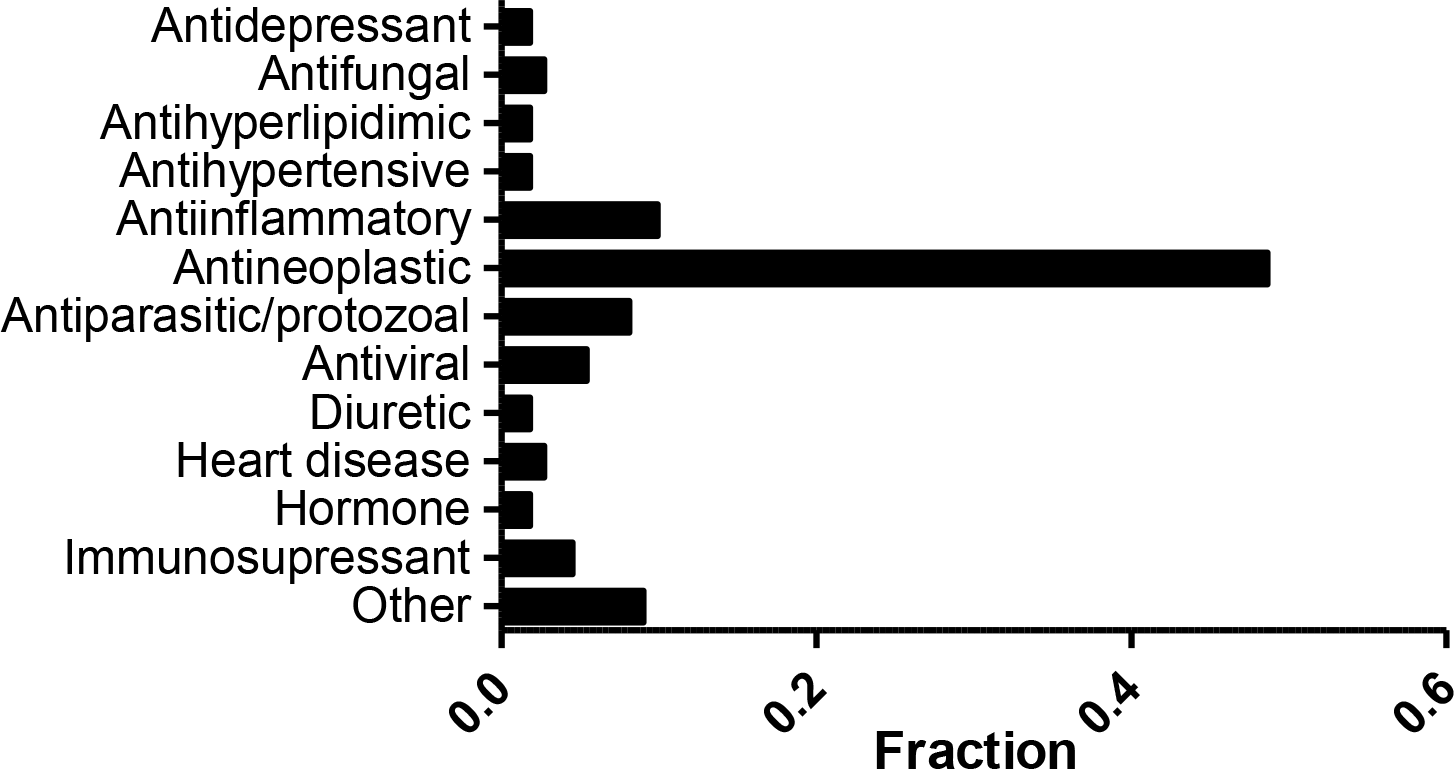
Active compounds with dosing/safety information in children Fraction of compounds that are active against one or more cell line and are part of the CPC. A total of 111 were grouped by indication. The category labeled as “Other” contains indications represented by 1 compound.

We have previously reported a comprehensive and growing resource, the Children's Pharmacy Collaborative (CPC) database, a listing of all drugs for which there are some dosing and safety information for children (0–18 years old) [4]. Of the 3,886 unique compounds included in the NPC and MIPE collections, 875 are also part of our CPC database. A total of 111 compounds that are active in our screen against one or more cell lines (and have minimal activity against Hh-Wt fibroblasts) are part of the CPC (Supplementary Table 8). Overall, the majority of active compounds approved for pediatric uses belong to the antineoplastic category (Figure 4). Among these agents we find drugs used in childhood cancers such as dactinomycin, vincristine, topotecan, etc. However, several drugs which are known to be effective against childhood cancers did not demonstrate the expected cytotoxicity in our screening. Drugs such as cyclophosphamide, platinating agents (carboplatin and cisplatin), and temozolomide, which either require activation *in vivo* (cyclophosphamide), are inactive as DMSO solutions (platinating agents), or have a short half-life *in vitro,* did not demonstrate activity in this system as might have been expected. Nine other anticancer agents, including sorafenib, vorinostat, and romidepsin did not meet criteria for activity in this assay.

In addition to antineoplastics, other compounds with cytotoxic activity against multiple pediatric cancer lines that are also present in the CPC include antiviral, antiparasitic/protozoal agents, as well as compounds with antiinflammatory and immunosuppressant indications as discussed above. Our screens indicated that cardiac glycosides such as digitoxin, digoxin, and oubain and β-adrenoreceptor agonists such as isoprotenerol and dobutamine, also display cytotoxic effects in cancer cell lines. These agents, traditionally used for the treatment of heart diseases, have also shown cytotoxic effects in cancer cell lines as well as *in vivo* xenograft models in numerous studies [31–38]. Among these agents (Supplementary Figure 6), digitoxin and dobutamine are included in the CPC and may merit follow up studies.

It is well established that three-dimensional (3D) cell cultures provide structural and cellular morphological complexity that can lead to different responses to therapeutic compounds compared to traditional monolayer cultures. Multicellular tumor spheroids is one of the most well characterized 3D culture systems, and combined with the ability to perform medium-throughput screening of 3D spheroids, it has become a cell-line platform to evaluate the therapeutic efficacy of anticancer agents [39–42]. Therefore, we sought to further validate compound activity in 3D multicellular tumor spheroids of pediatric cancer cell lines developed as *in vitro* solid tumor models. Specifically, we developed a multiparametric qHTS assay in 384-well format to monitor the effect of compounds on spheroid viability (Supplementary Figure 7). Briefly, cells were cultured in 384-well ultra-low attachment (ULA) plates for 48 hours to allow the formation of spheroids, which were subsequently treated with selected compounds in dose response. After 72 hour incubation, spheroid viability was first quantified via propidium iodide (PI, which stains dead cells) and Hoechst 3342 (which stains all cells) imaging and subsequently with CellTiter-Glo (3D). Importantly, the diameter of untreated spheroids increases during the time course of the assay, suggesting active cell proliferation (Supplementary Figure 8). We selected a representative cell line of each tumor type to grow as spheroids under ultra-low attachment (ULA) conditions. Specifically, we chose TC32 from Ewing's sarcoma, MG 63 (6-TG R) from osteosarcoma, RD from rhabdomyosarcoma, SK-N-SH from neuroblastoma, and Daoy and SJ-GBM2 from CNS tumors of the medulloblastoma and glioblastoma types, respectively. Neither cell line of the ATRT type formed spheroids under these conditions (data not shown). To identify new therapeutic options for pediatric solid cancers, we tested 60 compounds that were active in our phenotypic screen, 41 of which have safety/dosing profiles in children. The 60 compounds have mainly antineoplastic, anti-inflammatory, antiviral and antiparasitic/protozoal indications (Supplementary Table 9). Compound activity was determined based on the CellTiter-Glo parameter as before because of overall better assay statistics compared to imaging parameters (Supplementary Table 2). Nevertheless, compound potency determined using both CellTiter-Glo and imaging parameter is overall well correlated, except for MG 63 (6-TG R) and Daoy lines (Supplementary Figure 9).

**Figure 5:**
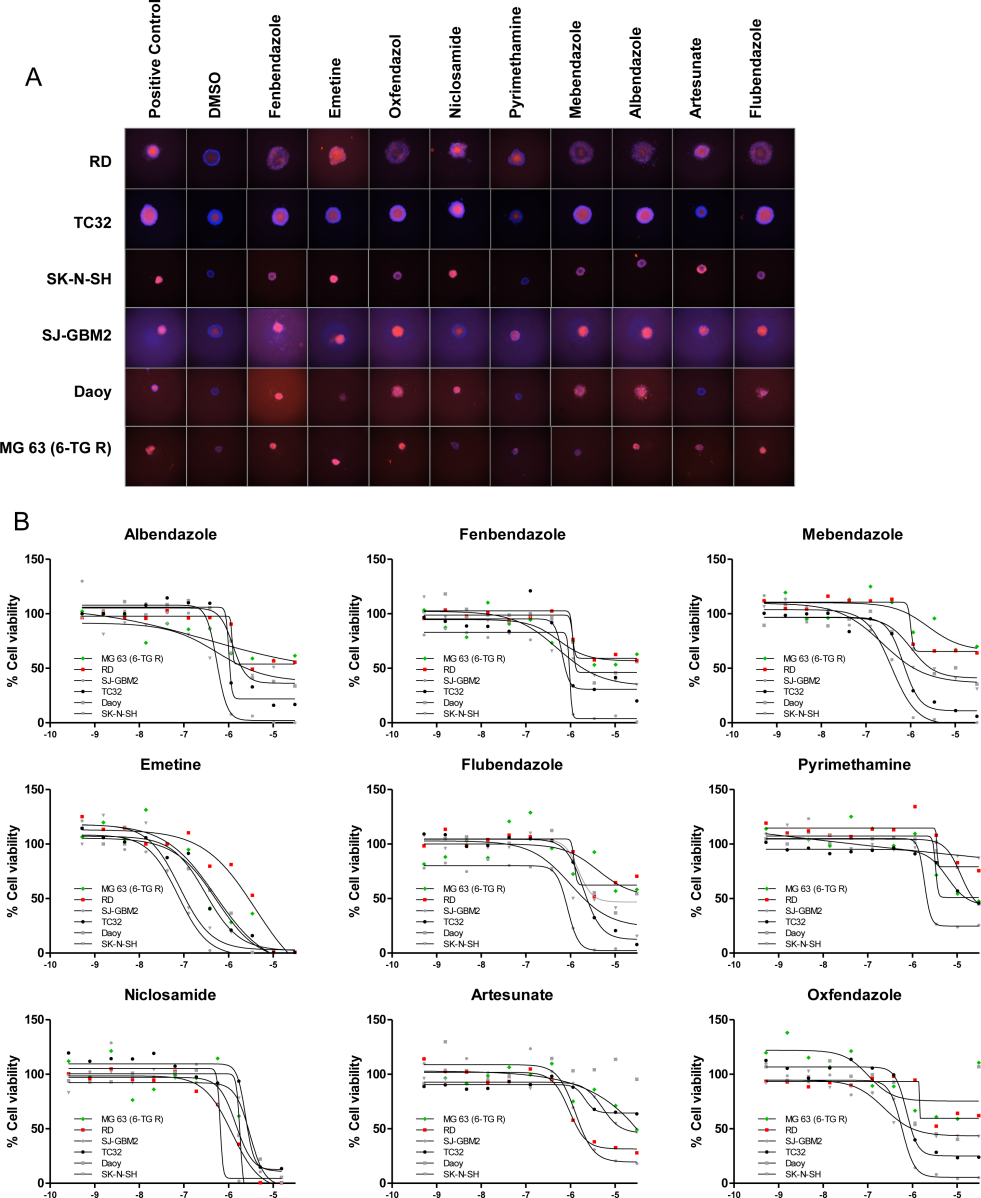
Antiparasitic/protozoal agents are active in 3D cultures (**A**) Representative images of spheroids treated with indicated agents (at 30 μM, except Niclosamide which was tested at 15 μM), for 72 hours and stained with Hoechst 3342 (blue) and PI (red). (**B**) Dose response curves of indicated agents treated as above but obtained by CellTiter-Glo (3D). X-axis indicates compound concentration in Log[M].

**Figure 6:**
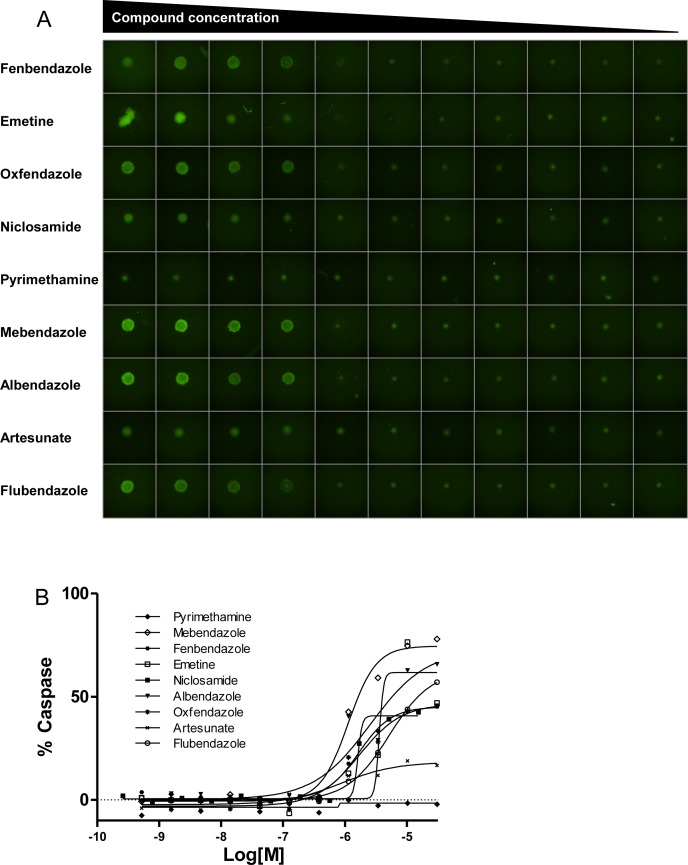
Antiparasitic/protozoal agents induce apoptosis in TC32 spheroids (**A**) Representative images of TC32 spheroids treated with indicated agents for 24 hours and stained with Caspase3/7-Alexa488 (green). Compounds were tested in dose response ranging from 30 μM to 0.5 nM, except Niclosamide, which was tested in the range of 15 μM to 0.25 nM. (**B**) Dose response curves of indicated agents. Data are represented as % Caspase-3/7 activation compared to staurosporine control.

A comparison of compound potency in 2D vs. 3D cultures indicated low to moderate correlation depending on the cell line, with several compounds displaying differential activity between formats. For instance, some compounds (particularly the antiviral acyclovir and the PARP inhibitor veliparib) showed cytotoxicity in 3D cultures despite being inactive (no cytotoxicity at maximum concentration tested or potency >10 μM) in the corresponding monolayer culture. Conversely, other compounds that showed cytotoxic activity in 2D monolayers were inactive (no cytotoxicity at maximum concentration tested or potency >10 μM cutoff) in 3D spheroid cultures. Among these, 7 compounds including the antiinflammatory agents ibuprofen, mometasone, and deflazacort did not show activity in any of the 3D cultures tested (Supplementary Figure 10). The remaining compounds reduced the viability of spheroids of at least one cell line, with 17 compounds displaying pan-activity across all 6 spheroid types. Pan-actives include not surprisingly antineoplatics agents such as adriamycin, bortezomib, and quisinostat but also the cardiotonic glycoside digitoxin and the antiparasitics emetine and niclosamide. In fact, all antiparasitic/protozoal agents tested show cell viability effects in 3D cultures (Figure 5). To determine if the cytotoxic effect elicited by antiparasitic/protozoal was due to apoptosis-mediated cell death, we quantified caspase-3/7 activation in TC32 spheroids using the CellEvent Caspase-3/7 green detection imaging reagent (Figure 6A). We found that all tested compounds induced strong caspase-3/7 activation with the exception of pyrimethamine (Figure 6B), which agrees with the lack of activity in the viability assay (Figure 5). Niclosamide and artesunate showed moderate caspase activity. In the case of artesunate, its effect was also in agreement with the viability assay; however, niclosamide was clearly active in the viability assay, indicating that it might elicit cell death via additional mechanisms. Most of these antiparasitic agents are included in the CPC and hence constitute good candidates for follow up *in vivo* studies in children.

## DISCUSSION

In contrast to hypothesis-driven testing of small numbers of compounds against childhood cancers, such as that performed by the NCI's PPTP [43], phenotypic HTS can provide information on large numbers of drugs and cell types in short periods of time. Moreover, many repurposed drugs act by apparently different mechanisms for their different indications, arguing for broad, mechanism-agnostic drug screening. In this study, we report the quantitative profiling of 19 pediatric cancer cell lines against a collection of ∼3,800 approved and investigational drugs. We identified and validated over ∼500 compounds with cytotoxic activity against one or more cell lines. Strengthening our approach, the majority of active compounds found in the screen have known antineoplastic indications or are being investigated for oncology purposes. However, some active hits have not generally been recognized as having activity against pediatric and/or adult cancer cell lines. Among the latter, we find compounds used as antiparasitics, antiprotozoals, antiinflammatories as well as agents for the treatment of heart diseases. We further validated the activity of 53 compounds in 3D tumor models of pediatric cancers using a multiparametric qHTS assay. Specifically, we found that antiparasitics reduce TC32 cell viability via induction of apoptosis.

Importantly for the intended pediatric repurposing, over 100 active compounds identified in our screen (∼50% of which were also validated in 3D spheroid assays) are also included in our CPC database. Some of these drugs, such as the immunosupressants primecolimus, mycophenolic acid and related mycophenolate mofetil have been used in children and/or adolescents for very different primary indications and if preclinical efficacy is verified by other assays (such as *in vivo* xenograft models), they should represent attractive candidates for phase II clinical trials. Antiparasitic such as mebendazole and difluoromethylornithine (DFMO) are currently in clinical trials for pediatric brain tumors and neuroblastomas. Our findings further indicate that antiparasitics could have therapeutic potential for a broad range of tumor types.

Our study has several limitations. Most importantly, although our extensive quantitative profiling of nearly four thousand drugs generated over half a million data points, we studied a relatively small number of cell lines of several tumor types, which particularly limits the identification of tumor-specific agents. In addition, paired control cell lines for each tumor type might better reflect broad vs. cancer-specific compound activity. For the primary and secondary screens, the assay readout is only a proxy for cell viability and orthogonal readouts might strengthen these findings, as seen in our 3D spheroid validation assay. Finally, efficacy in other biologically relevant models of childhood cancer, such as xenograft studies, should be tested to corroborate our findings.

We conclude that phenotypic qHTS is a valuable approach to identify new compounds and classes of drugs which merit additional attention for the treatment of pediatric solid tumors. In concert with informatics approaches, qHTS is useful for prioritizing drugs that already have safety profiles in children further facilitating drug development for pediatric solid tumors. The dataset generated here, along with the CPC, is fully available to the scientific community, and we hope that it will serve as valuable resource to accelerate drug repurposing.

## MATERIALS AND METHODS

### Cell lines and culture conditions

As shown in Table 1 and Supplementary Table 1, cell lines were obtained from the National Cancer Institute's Pediatric Preclinical Testing Program (NCI, PPTP) [44], maintained at Nationwide Children's Hospital, Columbus, OH and from laboratories at the Lineberger Comprehensive Cancer Center, University of North Carolina (courtesy of Drs. Bernard Weissman and Ian Davis). Control cell line Hh-Wt-fibroblasts were obtained from Coriell Institute (GM02153). Cell lines were cultured in the indicated media composition and maintained in a 37°C incubator with 5% CO_2_ and under a humidified atmosphere. DMEM, EMEM, IMDM, RPMI 1640, L-Glutamine, Insulin-Transferrin-Selenium-G, Pen/Strep (100 U/mL penicillin and 100 μg/mL streptomycin) were obtained from Thermo Fisher Scientific; McCoy's 5A Medium Modified was obtained from American Type Culture Collection; FBS was obtained from GE Healthcare Life Sciences. All cell lines were authenticated by short-tandem repeat (STR, 10 loci) profiling and routinely tested for mycoplasma contamination using MycoAlert mycoplasma detection kit (Lonza).

### Compounds

The NIH Chemical Genomics Center (NCGC) Pharmaceutical Collection (NPC) contains 2,816 compounds [21]. Of these, 50% are approved by the United States Food and Drug Administration (FDA). The remaining drugs are in use in other countries and/or have been tested in clinical trials against a range of diseases. The MIPE (Mechanism Interrogation Plate) collection contains 1,912 oncology-focused agents, many of which also have been approved for human use or are under clinical trials [18–20].

### qHTS cell viability assay

Cells were assayed in growth media at a density of 500–1,000 cells/well. Five μL of cells were dispensed into 1,536-well, white, solid-bottom, TC-treated plates (Greiner Bio One) using a Multidrop dispenser and incubated at 37°C, 5% CO_2_, under a humidified atmosphere for 5 hours. Twenty three nL of compounds and controls (neutral control DMSO or positive control bortezomib at final concentration of 2.3 μM) were subsequently transferred by Kalypsys pintool. For primary screens, the MIPE collection was tested at 11-point dilutions (final concentration range from 0.78 nM to 46 μM) and the NPC collection at 8-point dilutions (final concentration range from 0.59 nM to 46 μM). Compounds tested in follow-up screens were assayed at 11-point dilutions (final concentration range from 0.78 nM to 46 μM). Cells were incubated for 48 hours, followed by addition of 3 μL of CellTiter-Glo (Promega), then after a ∼15 minute incubation at RT, samples were analyzed for luminescence intensity using a ViewLux High-throughput CCD imager equipped with clear filters.

### qHTS viability assay in 3D cultures

Cells were assayed in phenol red-free growth media at a density of 1,000 cells/well, with the exception of Daoy, and SH-N-SH which were assayed at 1,500 cells/well. Thirty μL of cells were dispensed into 384-well, ULA, round bottom plates (Corning) using a Multidrop dispenser. Plates were spun down at 1,000 rpm for 1 min and incubated at 37°C, 5% CO_2_, under a humidified atmosphere for either 24 (Daoy) or 48 hours (all other cell lines) to allow spheroid formation. Ninety two nL of compounds and controls (neutral control DMSO or positive control bortezomib at final concentrations of 0.5 μM) were subsequently transferred via GNF Pintool. Plates were sealed using a breathable seal (Diversified Biotech, Dedham, MA) and incubated for 72 hours at 37°C. Spheroid viability was first determined by high-content imaging using Hoechst 33342 and Propidium Iodide (PI, ThermoFisher Scientific), followed by addition of CellTiter-Glo 3D (Promega). Specifically, 3 μL/well of PBS containing a 1:500 dilution of both dyes were dispensed using Multidrop. Dyes were incubated for 1 hour before imaging using the Celigo Image Cytometer. Then, 20 μl of CellTiter-Glo 3D were added per well. Plates were incubated for 30 min at RT on a VWR microplate shaker (300 rpm) and analyzed for luminescence intensity using a ViewLux High-throughput CCD imager equipped with clear filters.

Image analysis was done using the Celigo Tumorsphere 1+2+3 application software as previously described [45]. Briefly, spheroids were first identified using bright-field image segmentation, and then the fluorescent intensities of PI and Hoechst 3342 were obtained using red (EX: 531/40 nm, EM: 629/53 nm) and blue (EX: 377/50 nm, EM: 470/22 nm) filters, respectively. The data were exported to Excel, and the average fluorescent intensities were used to calculate the ratio of PI over Hoechst, which was used to quantify viability. Percent activity based on neutral and positive control bortezomib was derived and fitted as described in the qHTS data analysis and statistics section below.

### qHTS apoptosis assay in 3D cultures

Apoptosis assays were carried out as above with the difference that compounds were incubated for 24 hours before addition of CellEvent Caspase-3/7 green detection reagent (TheremoFisher Scientific). Staurosporine at final concentrations of 3 μM was used as positive control. Spheroids were imaged using bright-field and green (EX: 483/32 nM, EM: 536/40 nM) filters and analyzed with the Celigo Tumorsphere 1+2 application software. Spheroids were first identified using bright-field segmentation, and the average fluorescent intensity of the green channel was used to quantify caspase activation as percent activity based on neutral and positive controls (see qHTS data analysis and statistics below).

### qHTS data analysis and statistics

The screening data was analyzed using software developed internally in NIH Chemical Genomics Center. Data from each assay were normalized plate-wise to corresponding intra-plate controls as described previously [46]. The same controls were also used for the calculation of the Z’ factor index for each assay. The Z’ factor, a measure of assay quality control, was determined by Z′ = 1 – (3 × SD_positive_ + 3 × SD_neutral_)/(Mean_positive_ – Mean_neutral_) where SD is the standard deviation [47]. Supplementary Table 2 shows S:B and Z’ values for all primary and follow-up screens. Percent activity was derived using in-house software (http://tripod.nih.gov/curvefit/). Dose-response curves were classified as described previously [22]. All concentration–response curves (CRC) were fitted, and IC_50_ were calculated with the GraphPad Prism^®^ software (GraphPad, San Diego, CA). Data sets were generated for each cell line against each of the drugs which included an activity score based on potency (IC_50_), maximum responses, curve classes, fit log IC_50_, and fit Hill slopes. Compounds exhibiting high-quality CRCs (class -1 and -2), and CellTiter-Glo signals six standard deviations below the population of neutral control wells, were considered active. Activity was further refined by imposing a cutoff IC_50_ of <10 μM, and Maximal response >65%. In the case of spheroid assays, each compound was tested in triplicate. Compounds were considered active when 2 or 3 replicates passed the above cutoffs.

### Clustering of compounds by activity profiles

Compounds were clustered hierarchically within TIBCO Spotfire 6.0.0 based on their activity outcomes from the primary or follow-up screen across cell lines. Either compound's potency or compound's AUC (Area Under the Curve, calculated based on the qHTS data analysis and curve fittings), were utilized for clustering. In the heatmap, darker color indicates compounds that are more potent and efficacious, i.e. high-quality actives, and lighter color indicates for less potent and efficacious compounds. If a compound didn't show any activity in an assay, it was highlighted as white in the heatmap. In potency-based heatmaps, inactive compounds that have no IC_50_ values determined were showed up as grey.

## SUPPLEMENTARY MATERIALS FIGURES AND TABLES



















## Abbreviations

qHTS: quantitative high-throughput screening
CRCs: concentration response curves
CPC: Children's Pharmacy Collaborative database
3D: three-dimensional
EWS: Ewing's sarcoma
CNS: central nervous system
OS: osteosarcoma
ATRT: atypical teratoid rhabdoid tumor
RMS: rhabdomyosarcoma
HDAC: histone deacetylase
ITK: Interleukin-2-inducible T cell kinase
Jak2: Janus kinase 2
PI3K: phosphoinositide 3-kinase inhibitor
Hh-Wt: Human wild-type
mTOR: mammalian target of rapamycin
NSAID: non-steroidal anti-inflammatory drug
NPC: NIH Chemical Genomics Center (NCGC) Pharmaceutical Collection
MIPE: Mechanism Interrogation Plate
NB: Neuroblastoma.
