# Supplementary material for: Quantitative high-throughput phenotypic screening of pediatric cancer cell lines identifies multiple opportunities for drug repurposing
Source: Oncotarget. 2017 Dec 19;9(4):4758–72. doi: 10.18632/oncotarget.23462 (PMC5797010; doi:10.18632/oncotarget.23462)
Supplement: Supplementary file 1 [file oncotarget-09-4758-s001.pdf]

Quantitative high-throughput phenotypic screening of pediatric cancer cell lines identifies multiple opportunities for drug repurposing

SUPPLEMENTARY MATERIALS

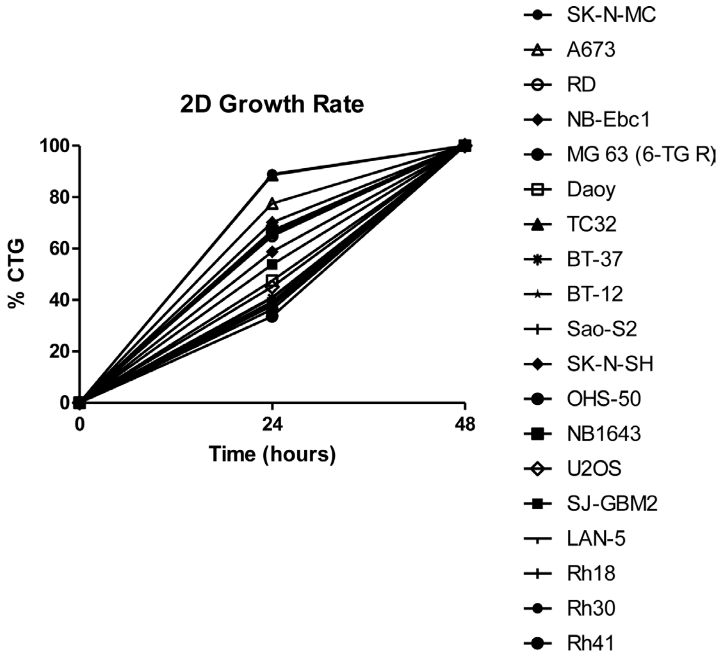

Supplementary Figure 1: Growth rate of 19-cell line panel during time course of assay. For each cell line, viability was determined using Cell Titer-Glo reagent at 0, 24 and 48 hr after plating into 1,536-well plates. Growth rate is represented as percent CTG signal normalized to 0 hr (0%) and 48 hr (100%).

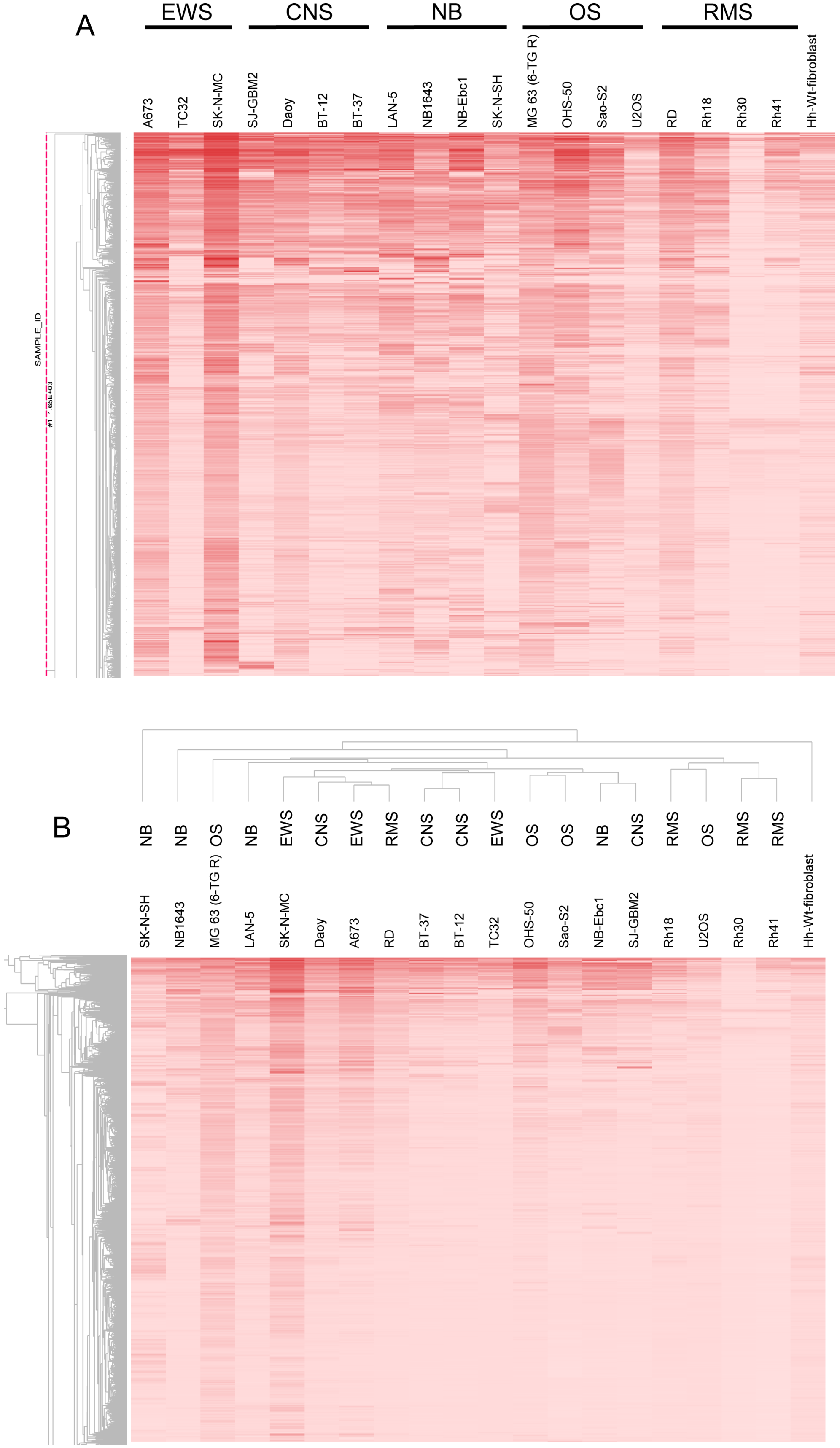
A
EWS
CNS
NB
OS
RMS
A673
TC32
SK-N-MC
SJ-GBM2
Daoy
BT-12
BT-37
LAN-5
NB1643
NB-Ebc1
SK-N-SH
MG 63 (6-TG R)
OHS-50
Sao-S2
U2OS
RD
Rh18
Rh30
Rh41
Hh-Wt-fibroblast
SAMPLE_ID
B
NB
NB
OS
NB
EWS
CNS
EWS
RMS
CNS
CNS
EWS
OS
OS
NB
CNS
RMS
OS
RMS
RMS
SK-N-SH
NB1643
MG 63 (6-TG R)
LAN-5
SK-N-MC
Daoy
A673
RD
BT-37
BT-12
TC32
OHS-50
Sao-S2
NB-Ebc1
SJ-GBM2
Rh18
U2OS
Rh30
Rh41
Hh-Wt-fibroblast

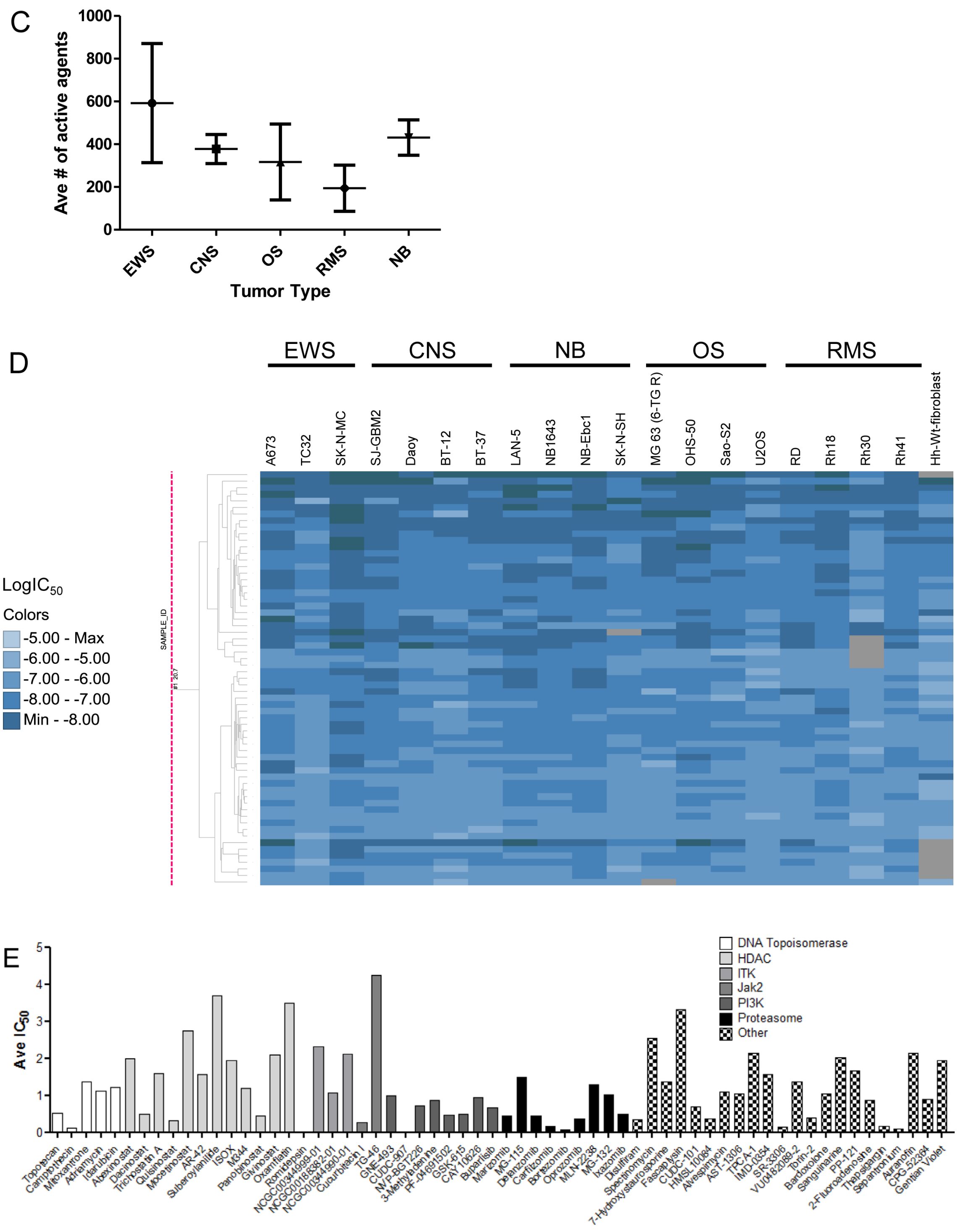

Supplementary Figure 2: qHTS of NPC and MIPE collections identifies agents that inhibit the proliferation of pediatric cancer cell lines. (**A**) Hierarchical clustering of compound activity in the primary screen rendered by tumor type. Compound activity is based on AUC (Area Under the Curve) values. AUC values are colored red, with darker color indicating compounds that are more potent and efficacious. (**B**) Hierarchical clustering of cell lines rendered by compound activity (calculated as AUC values) in the primary screen. AUC values are colored red, with darker color indicating compounds that are more potent and efficacious. (**C**) Average number of active compounds per tumor type. (**D**) Hierarchical clustering of pan-active compounds (active in 17 cell lines or more) in the primary screen based on potency. LogIC_{50} values are colored in blue, with darker color indicating more potent compounds. Inactive compounds that have no IC_{50} values determined are shown in grey. (**E**) Target-based analysis of pan-active compounds. For each compound, the average IC_{50} across all cell lines in shown.

A

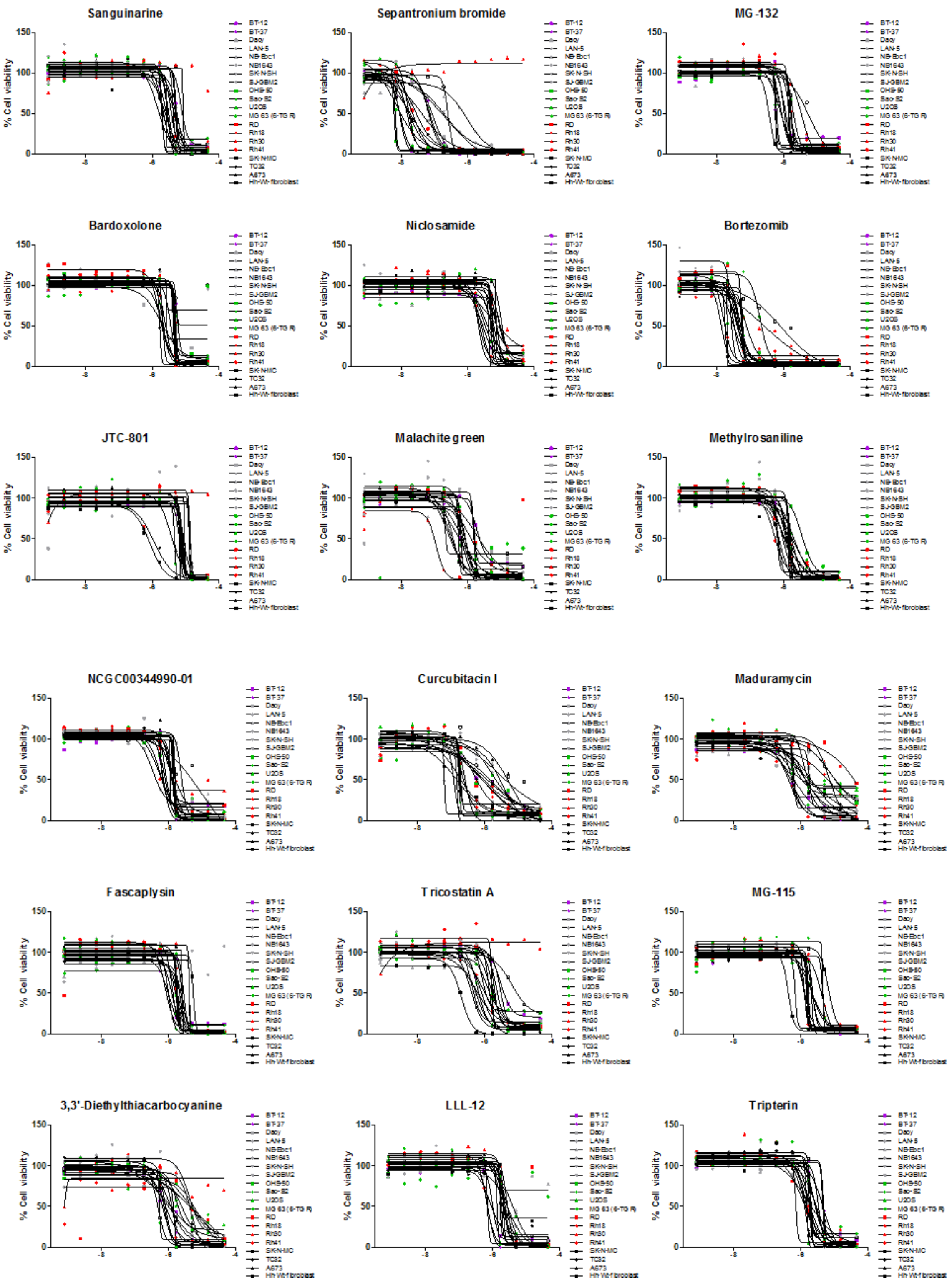
Sanguinarine
Sepantronium bromide
MG-132
Bardoxolone
Niclosamide
Bortezomib
JTC-801
Malachite green
Methylrosaniline
NCGC00344990-01
Curcubitacin I
Maduramycin
Fascaplysin
Tricostatin A
MG-115
3,3'-Diethylthiacarbocyanine
LLL-12
Tripterin
% Cell viability

B

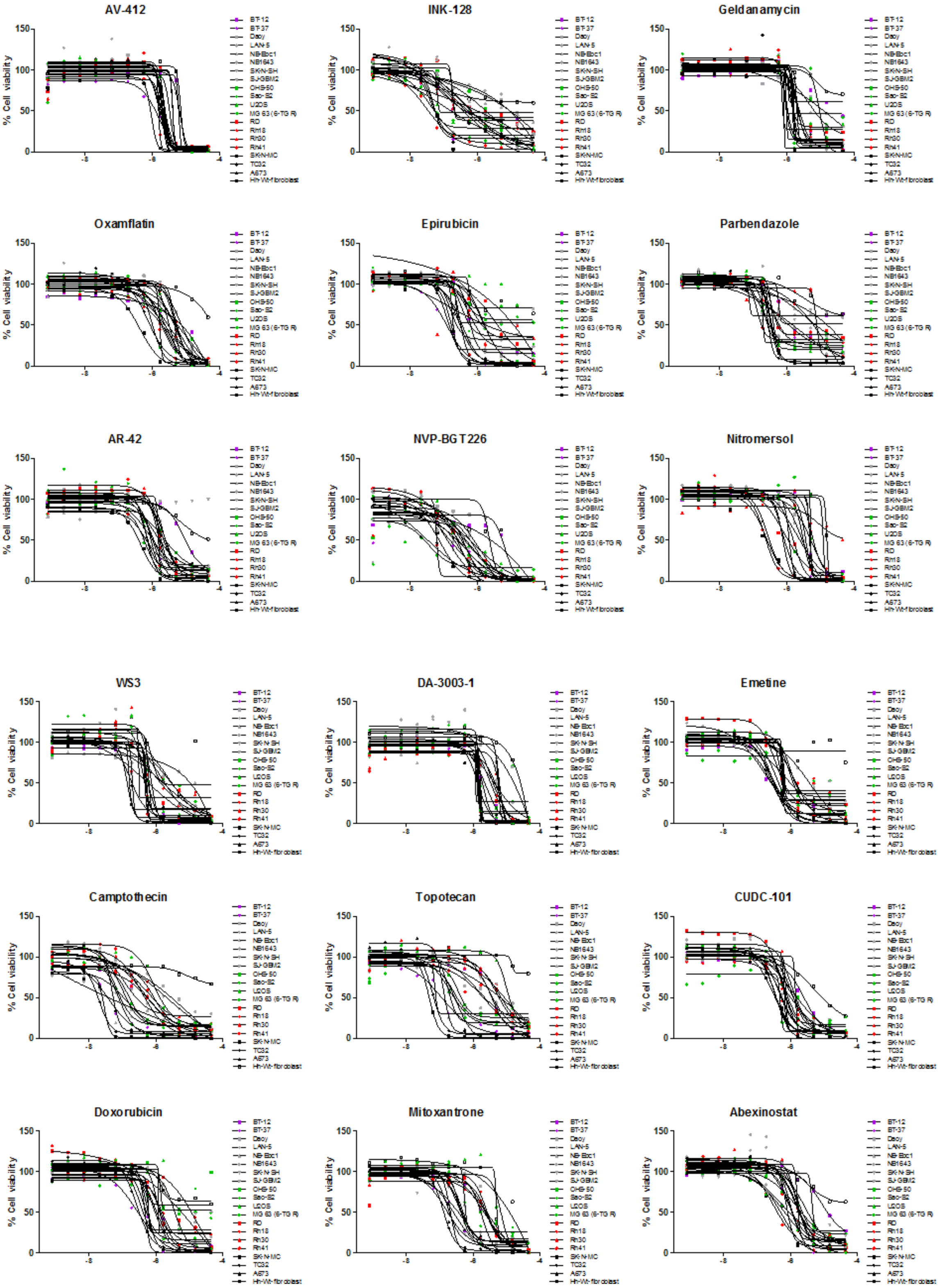
AV-412
INK-128
Geldanamycin
Oxamflatin
Epirubicin
Parbendazole
AR-42
NVP-BGT226
Nitromersol
WS3
DA-3003-1
Emetine
Camptothecin
Topotecan
CUDC-101
Doxorubicin
Mitoxantrone
Abexinostat
% Cell viability
BT-12
BT-37
Daoy
LAN-5
NB-Ebc1
NB1643
SK-N-SH
SJ-GBM2
OHS-50
Saos-2
U2OS
MG 63 (6-TG R)
RD
Rh18
Rh30
Rh41
SK-N-MC
TC32
A673
Hh-Wt-fibroblast

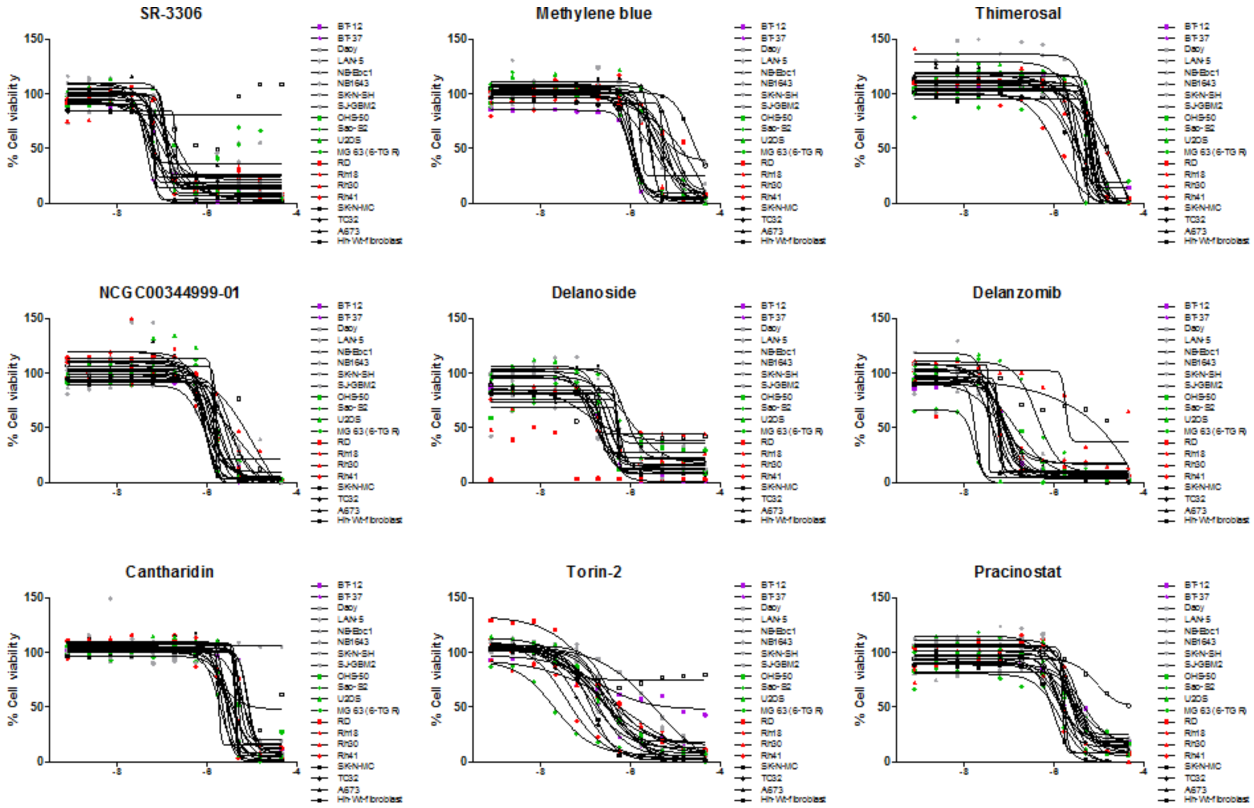

Supplementary Figure 3: Dose-response curves of pan-active compounds identified in follow-up screens. (**A**) Dose response curves of pan-active compounds with activity against Hh-Wt-fibroblasts. (**B**) Dose response curves of pan-active compounds with minimal activity against Hh-Wt-fibroblasts. X-axis indicates compound concentration in Log[M].

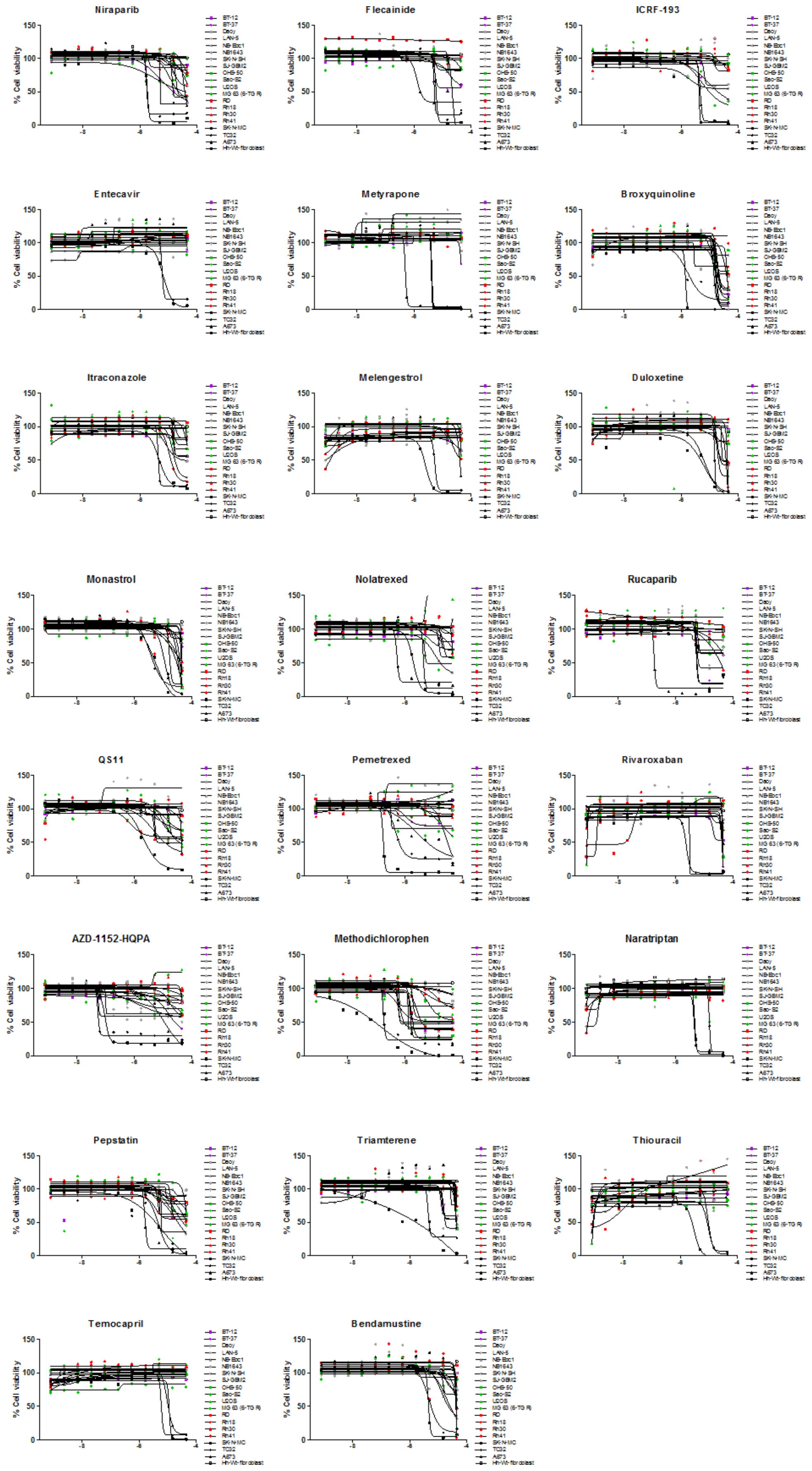

Supplementary Figure 4: Dose-response curves of EWS-specific active compounds identified in follow-up screens. X-axis indicates compound concentration in Log[M].

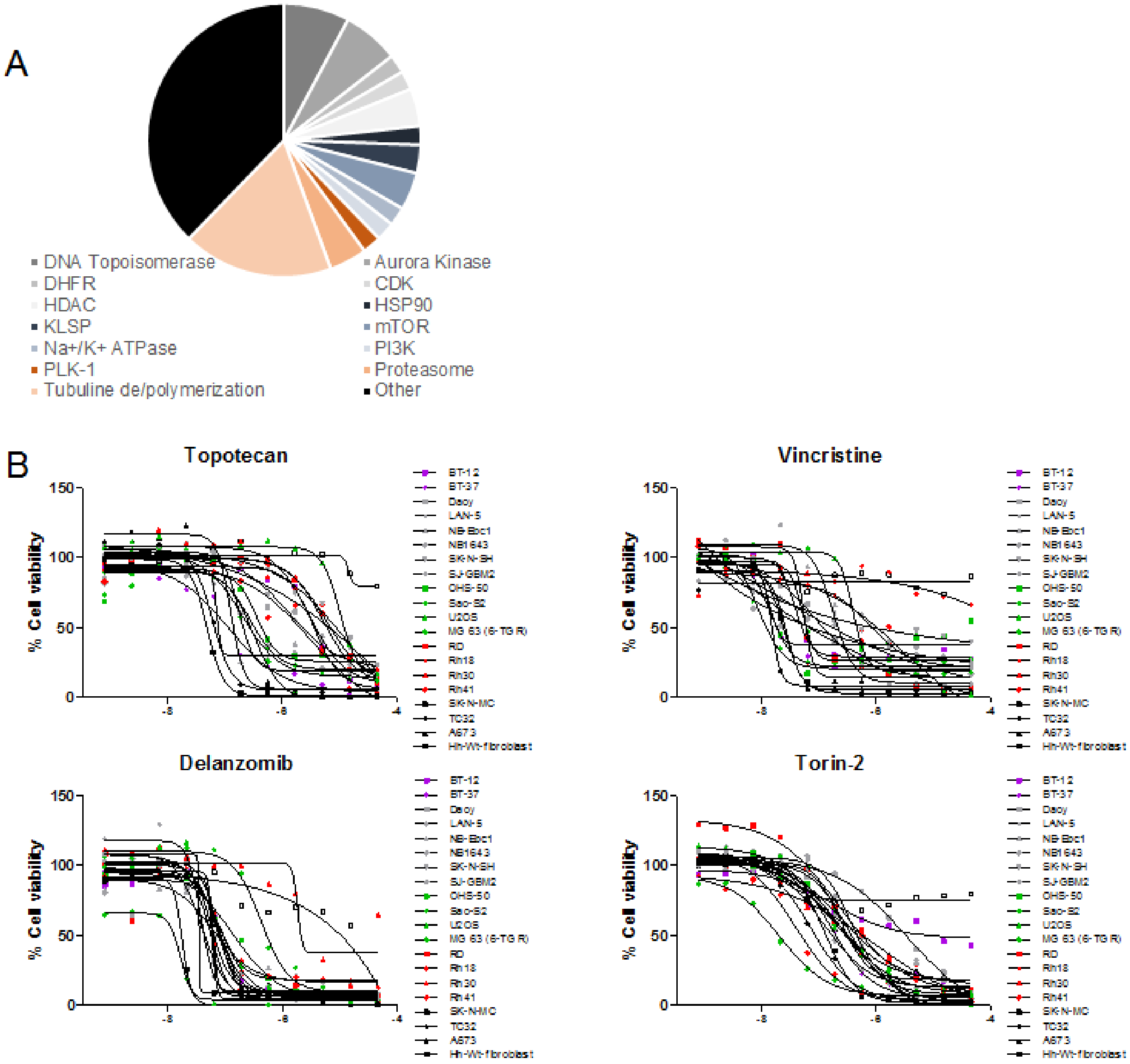

Supplementary Figure 5: Compounds with potent activity (<100 nM) identified in the screen. (**A**) Target-based analysis of potent compounds. (**B**) Dose response curves of potent compounds. Exemplified are four compounds with antineoplastic indication. X-axis indicates compound concentration in Log[M].

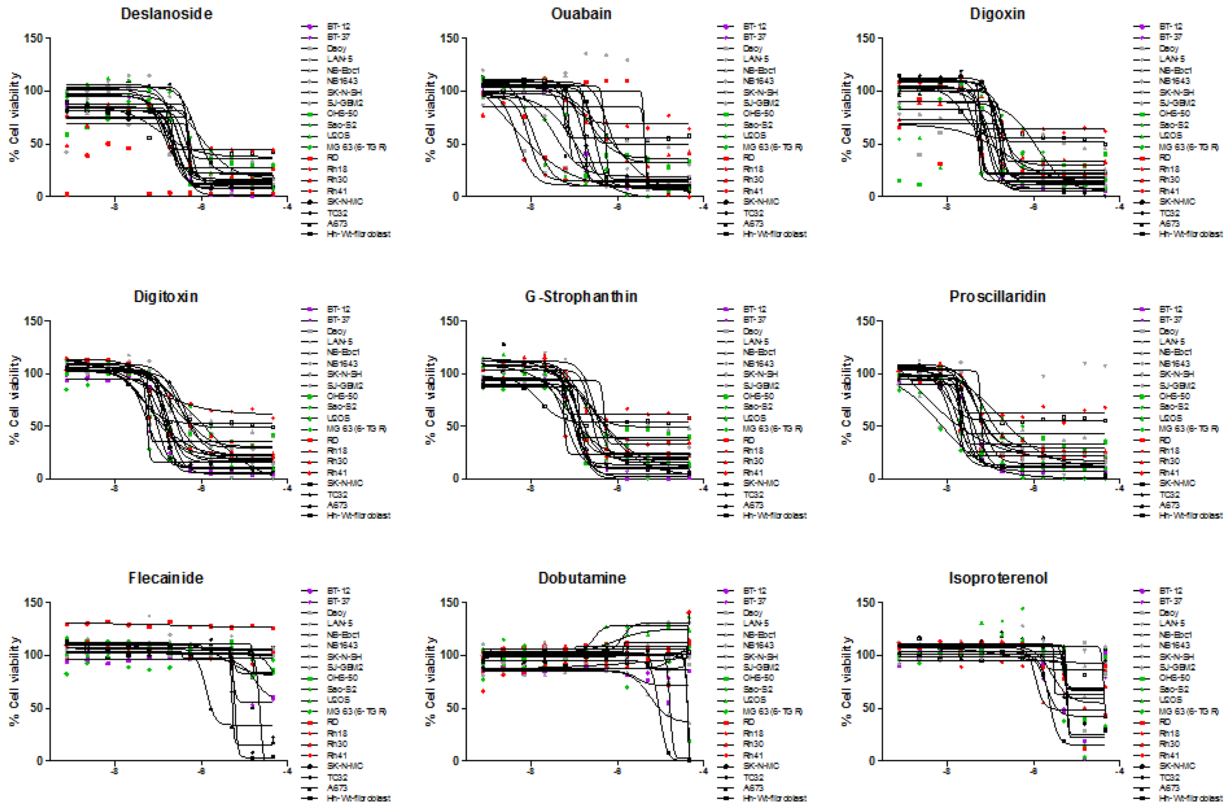

Supplementary Figure 6: Dose-response curves of agents traditionally used for heart ailments. Digoxin, isoproterenol, and dobutamine are include in the CPC. X-axis indicates compound concentration in Log[M].

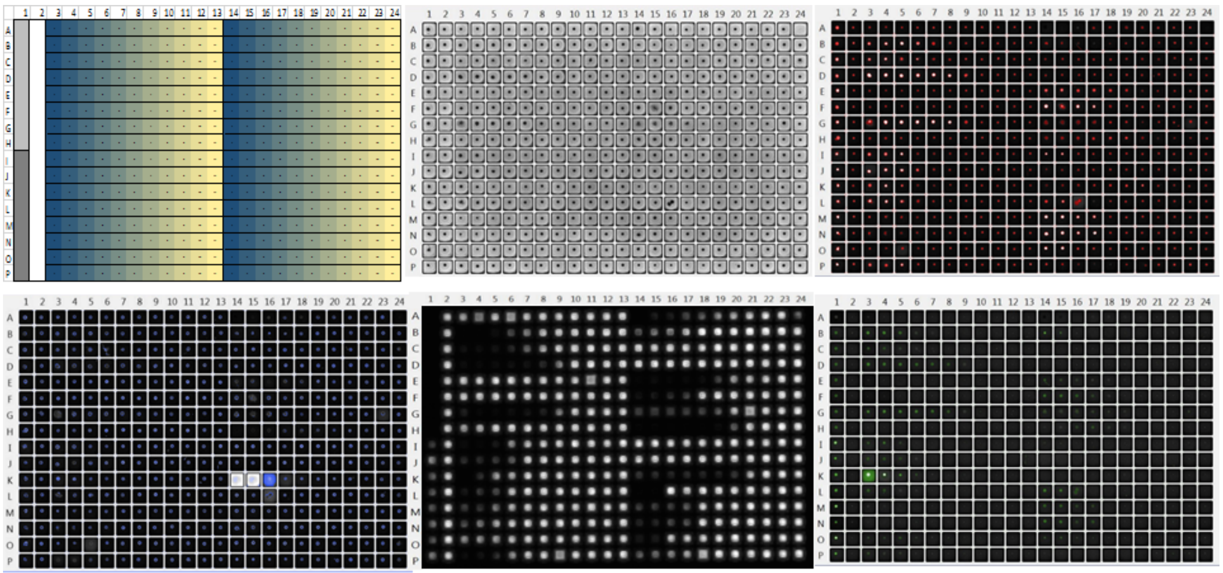

Supplementary Figure 7: Validation strategy in 3D cultures. Spheroids grown on 384-well were treated with compounds for 48 hr. Top-left: Each compound was assayed at 11-point doses arranged row-wise (high-to-low concentration depicted by blue-to-yellow gradient) and activity was compared to neutral control DMSO (column 2) and positive controls bortezomib (column 1, row A-H) and staurosporine (column 1, row I-P). Viability was subsequently assayed at 48 hr by bright-field (top-middle), PI (top-right) and Hoechst 3342 (bottom-left) imaging, followed by CellTiter-Glo (bottom-middle). Bottom-right: TC32 spheroids were assayed for apoptosis induction at 24 hr by imaging caspase signal.

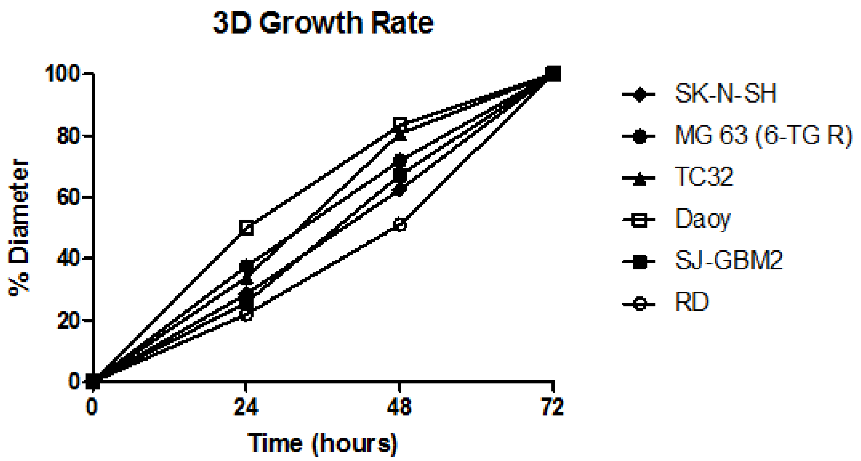

Supplementary Figure 8: Growth rate of 3D cell cultures during time course of viability assay. Cells were plated into 384-well ULA plates and allow to form spheroids for either 24 (Daoy) or 48 (remaining cell lines) hr. Subsequently, diameter was measured using brightfield segmentation (Celigo software) at 0, 24, 48 and 72 hr. Growth rate is represented as average percent diameter (n=16) normalized to 0 hr (0%) and 72 hr (100%).

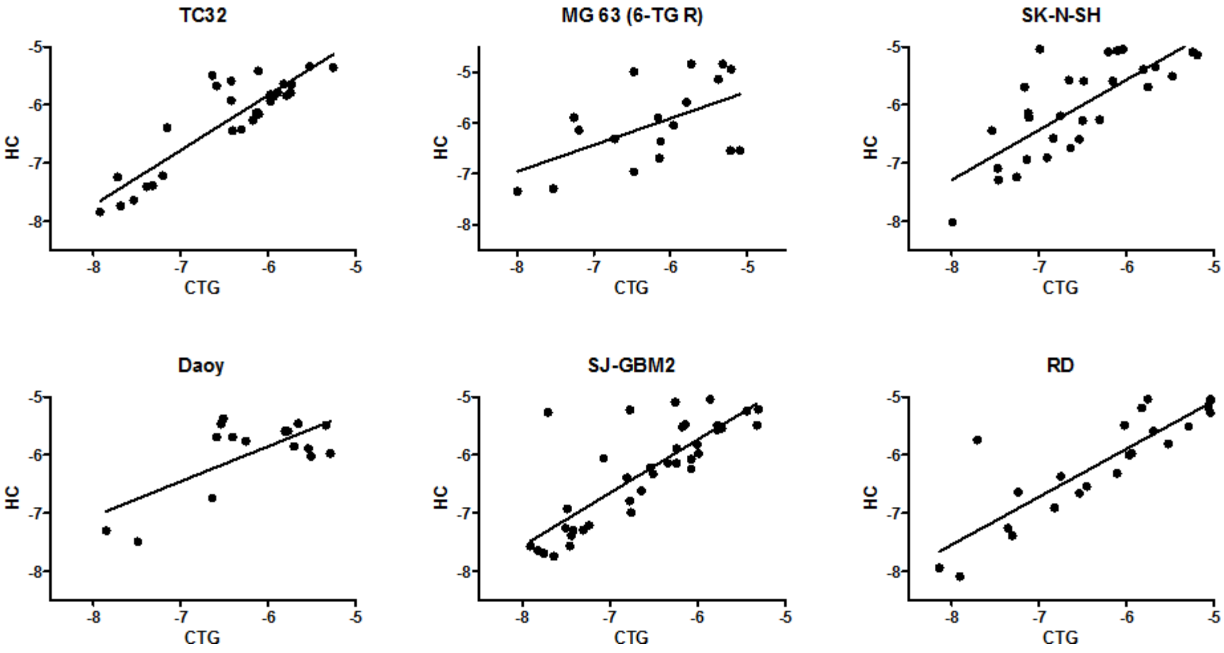

Supplementary Figure 9: Compound potency correlation between parameters in 3D cultures. Correlation plots of compound Log(IC_{50}) values obtained with high-content imaging (HC) and CellTiter-Glo (CTG) assays for TC32 ($R^2 = 0.79$), MD 63 (6-TG R) ($R^2 = 0.30$), SK-N-SH ($R^2 = 0.58$), Daoy ($R^2 = 0.48$), SJ-GBM2 ($R^2 = 0.65$) and RD ($R^2 = 0.74$) spheroids. Only compounds that passed the activity criteria in both assay types are shown and were used to calculate correlation coefficients.

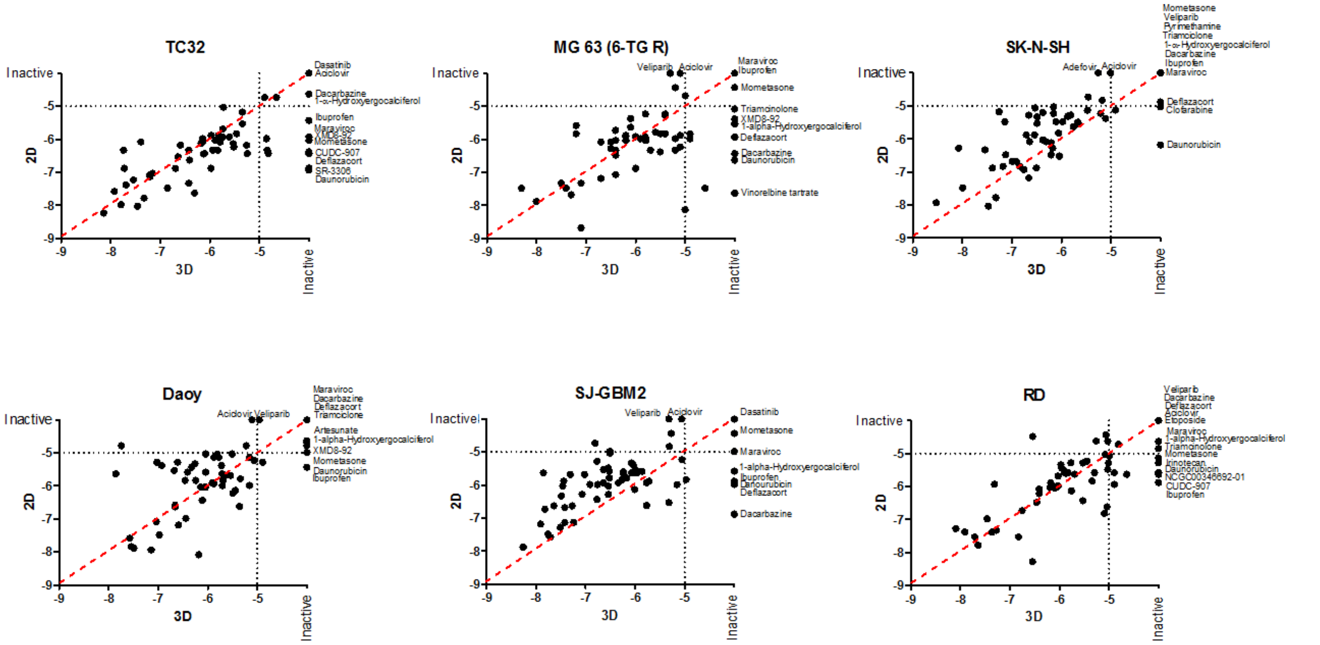

Supplementary Figure 10: Compound potency correlation between 2D monolayers and 3D spheroid cultures. Correlation plots of compound potency represented as Log(IC_{50}) values obtained in 2D (y-axis) and 3D (x-axis) cultures using CellTiter-Glo (CTG) readout. Red dotted line indicates perfect correlation and black dotted lines, indicate the 10 µM potency cutoff utilized to define active compounds. The name of inactive compounds for which no IC_{50} could be determined (no observed cytotoxicity at maximum concentration tested) in either or both culture formats are highlithed. Inactive compounds were not used to calculate correlations (R^2 values are 0.57, 0.50, 0.27, 0.51, 0.2 and 0.37 for TC32, SK-N-SH, MG 63 (6-TG R), RD, Daoy and SJ-GBM2, respectively).

Supplementary Table 1: Culture conditions

**Cell line**	**Culturing conditions**		
**Name**	**Media**	**FBS**	**Supplement**
A673	DMEM	10%	1% P/S
TC32	RPMI 1640	10%	1% P/S
SK-N-MC	EMEM	10%	1% P/S
SJ-GBM2	IMDM	20%	1% P/S, 1% L-Glut, 1% ITS-G
Daoy	EMEM	10%	1% P/S
BT-12	RPMI 1640	20%	1% P/S, 1% L-Glut, 1% ITS-G
BT-37	RPMI 1640	10%	1% P/S
LAN-5	RPMI 1640	10%	1% P/S
NB1643	RPMI 1640	10%	1% P/S, 1% L-Glut
NB-EBc1	IMDM	20%	1% P/S, 1% L-Glut, 1% ITS-G
SK-N-SH	DMEM	10%	1% P/S
MG 63 (6-TG R)	EMEM	HI, 10%	1% P/S
OHS-50	RPMI 1640	10%	1% P/S
Saos-2	McCoy's 5a	15%	1% P/S
U-2 OS	McCoy's 5a	10%	1% P/S
RD	DMEM	10%	1% P/S
Rh18	RPMI 1640	20%	1% P/S, 1% L-Glut, 1% ITS-G
Rh30	IMDM	20%	1% P/S, 1% L-Glut, 1% ITS-G
Rh41	IMDM	20%	1% P/S, 1% L-Glut, 1% ITS-G
Hh-Wt-fibroblasts	MEMalpha	15%	1% P/S

HI: Heat Inactivated; P/S: Penicillin/Streptomycin; L-Glut: L-Glutamine; ITS-G: Insulin-Transferrin-Selenium; IMDM: Iscove's Modified Dubelcco's Media; EMEM: Eagle's Minimum Essential Medium; DMEM: Dubelcco's Modified Eagle's Medium; RPMI: Roswell Park Memorial Institute; MEM: Minimum Essential Medium; FBS: Fetal Bovine Serum.

Supplementary Table 2: Assay performance. For each cell line, assay window (S:B or signal-to-background) and Z' factor are represented as mean values ± standard deviation calculated from 16, 18, 8 or 6x1,536-well plates in the MIPE, NPC, follow-up screens and 3D assays respectively. See Supplementary_Table_2

Supplementary Table 3: Compounds that passed the activity cutoff in follow up screens. See Supplementary_Table_3

Supplementary Table 4: Average compound activity by tumor type in the follow up screen. See Supplementary_Table_4

Supplementary Table 5: Indication of pan-active compounds. See Supplementary_Table_5

Supplementary Table 6: Indication of active compounds with potencies of <100 nM against at least one cell line. See Supplementary_Table_6

Supplementary Table 7: Indication of active compounds identified in the screen. See Supplementary_Table_7

Supplementary Table 8: List of active compounds in follow up screens that are part of CPC (pediatric uses). Potency in follow up screens is shown (Ave IC_{50}, SD and *n* = number of cell lines in which the compound was active). See Supplementary_Table_8

Supplementary Table 9: List of compounds tested in 3D viability assays. Potency in CellTiter-Glo assay is shown as Ave IC_{50}, SD and *n* = number of cell lines in which the compound was active. See Supplementary_Table_9
